# Supplementary material for: Targeting netrin‐1/DCC interaction in diffuse large B‐cell and mantle cell lymphomas
Source: EMBO Mol Med. 2016 Feb 1;8(2):96–104. doi: 10.15252/emmm.201505480 (PMC4734837; doi:10.15252/emmm.201505480)

Targeting netrin-1/DCC interaction in Diffuse Large-B and Mantle Cell Lymphoma.

Laura Broutier[1], Marion Creveaux[1]*, Jonathan Vial[1]*, Antonin Tortereau[1,2]*, Guillaume Chazot[1], Mark MacCarron[3], Sophie Léon[4], Céline Pangault[5], Nicolas Gadot[6], Amélie Colombe[4], Marie-Laure Boulland[5], Jonathan Blachier[1], Julien C. Marie[3], Alexandra Traverse-Glehen[7], Olivier Donzé[8], Catherine Chassagne-Clément[4], Gilles Salles[7], Karin Tarte[5], Patrick Mehlen[1$] and Marie Castets[1$].

APPENDIX

Table of content

- **Legends for Appendix Figures**
- **Appendix Figures (S1 to S2)**

Legends for Appendix Figures

Appendix Figure S1. Characterization of lymphoid tumoral hyperproliferations in DCC-D1290N mutant mice.

A. Frequence of lymphoid lesions in DCC mutant (n=29) and control (n=19) mice.

B. Clonal origin of B-cell lymphoma in DCC-mutant mice. Rearranged immunoglobulin VDJ sequences were amplified by PCR as described in "Materials and methods". The three expected control DJ_H bands are indicated by arrows on the left, based on the amplification of the spleen DNA of a lymphoma-free $DCC^{+/+}$ mouse.

C. Histological analysis of lymphoid proliferations in DCC control and mutant mice. High magnifications of sections from control spleen, low-grade FL and high-grade DLBCL stained with hematoxylin-eosin-safran are shown. w: white pulp, r: red pulp, cr: compressed red pulp.

Appendix Figure S2. Netrin-1 acts as a survival factor for ABC-DLBCL and MCL cell lines.

A. Expression of netrin-1 (dark colors) and DCC (bright colors) in lymphoma cell lines. Quantification was performed by Q-RT-PCR in 21 lymphoma cell lines. *HPRT* housekeeping gene was used as a standardization control. Results are presented relatively to netrin-1/DCC expression levels in Oci-Ly3. Histogram bars corresponding to ABC-DLBCL, MCL and GC-DLBCL are respectively colored in blue, grey and green. Crosshatched bars correspond to other B-cell types of lymphoma.

B-C. Efficiency of netrin-1 (B) and DCC (C) silencing by siRNA in Granta-519 transfected cells. Netrin-1 and DCC expressions are respectively decreased by 65.2% and 78.4% in Granta-519 transfected with specific siRNA as compared to those transfected with scrambled siRNA. Results of Q-RT-PCR are presented as mean+/-std of at least 3 independent quantifications, relatively to *HPRT* housekeeping gene expression levels.

D. Effect of net-1 mAb on netrin-1 expressing Oci-Ly10 cell density. Results are means+/-std indexed to control of four independent experiments. *: p=0.05; two-sided Mann-Whitney U-test.

E. Effect of net-1 mAb on induction of Oci-Ly10 cells apoptosis, detected by TUNEL staining. Left panel: representative images are shown. TUNEL positive cells are labelled in red. Nuclei are counterstained in blue by Hoechst staining. Right panel: quantification of one representative experiment out of three performed is exposed. Results are presented as percentage of TUNEL-positive cells per field, indexed to control mean. **: p=0.006; two-sided Mann-Whitney U-test.

F. Effect of net-1 mAb on netrin-1 negative SUDHL4 cell density. Results are means+/-std indexed to control of three independent experiments. p>0.05; two-sided Mann-Whitney U-test.

G. Caspase-3 activity in Granta-519 (left panel, grey shading) and OCI-Ly3 (right panel, blue shading) cells treated with net-1 mAb antibody or with an unrelated Ig-G1 antibody (Ctl), with or without addition of an excess of netrin-1 to reverse effects of netrin-1-interfering antibody. Results are means+/-std of at least three independent experiments. $^{\$}$: p=0.05; *: p=0.04; $^{\$\$}$: p=0.03; **: p<0.009 ; two-sided Mann-Whitney U-test.

H. Survival of OCI-Ly3 xenografted mice after treatment with net-1 mAb 20mg/kg (n = 30) or control antibody (n = 30). p<0.0001; Log-rank (Mantel Cox) test.

Appendix Figure S1

A

Lymphoid lesions	Percentage of cases	
	DCC^{WT}	DCC$^{mut/mut}$
**FL**	5.2%	**17.2%**
**DLBCL**	10.5%	**13.8%**
**Hyperplasia**	0	**13.8%**

B

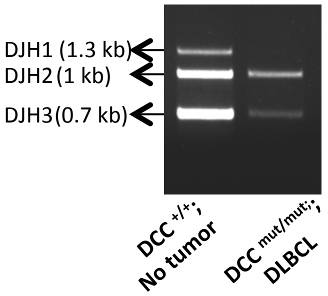

C

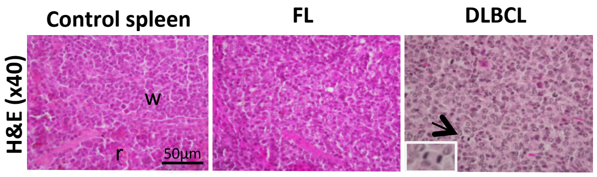

Appendix Figure S2

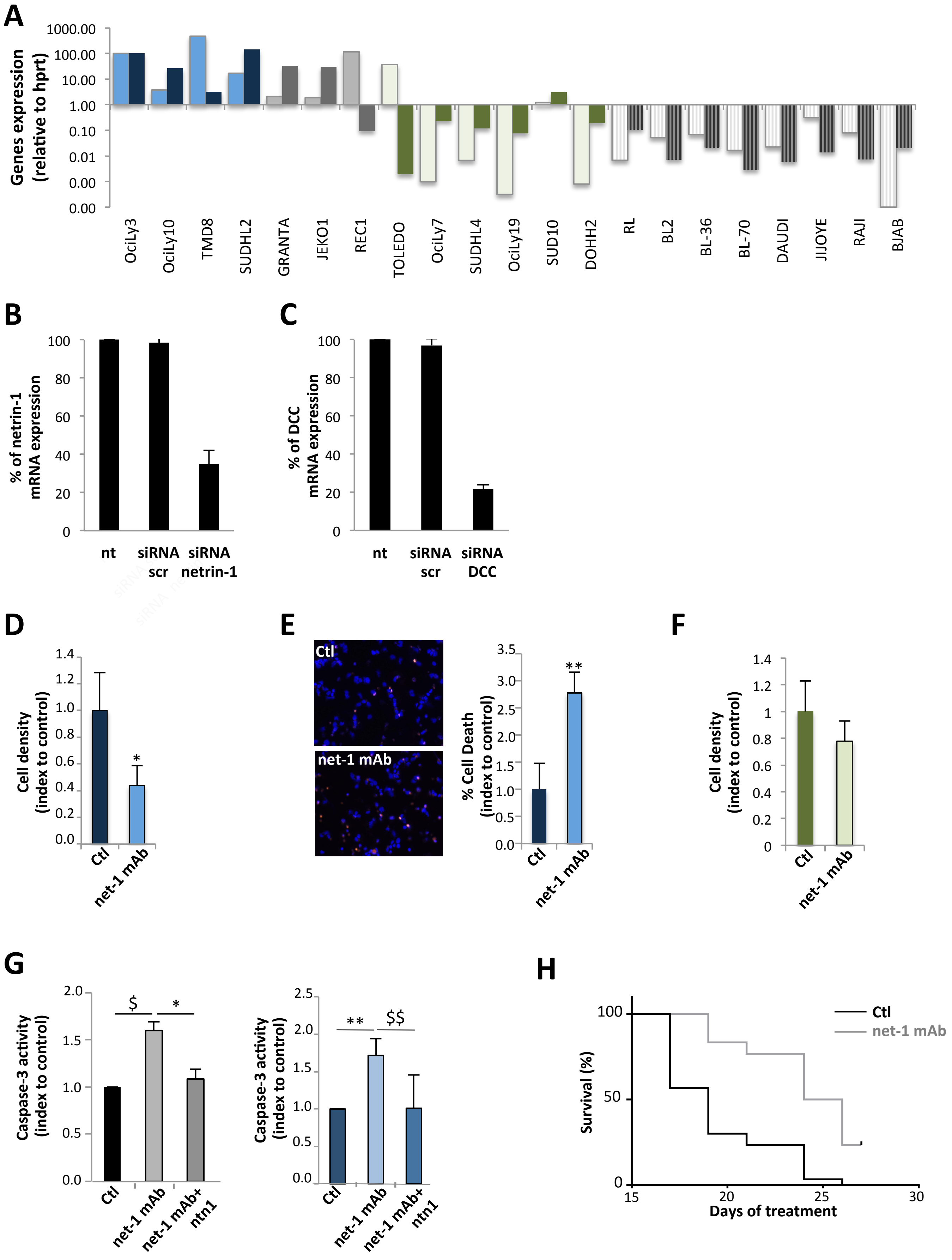

Supplement: Supplementary file 1 — Appendix [file EMMM-8-096-s001.pdf]
